# Characterization of Two Distinct Lymphoproliferative Diseases Caused by Ectopic Expression of the Notch Ligand DLL4 on T Cells

**DOI:** 10.1371/journal.pone.0084841

**Published:** 2013-12-27

**Authors:** Huizhong Xiong, Antonio Maraver, Jo-Ann Latkowski, Tanya Henderson, Karni Schlessinger, Yi Ding, Jie Shen, Carlos E. Tadokoro, Juan J. Lafaille

**Affiliations:** 1 Kimmel Center for Biology and Medicine at the Skirball Institute, New York University School of Medicine, New York, New York, United States of America; 2 The Sackler Institute of Graduate Biomedical Sciences, New York University School of Medicine, New York, New York, United States of America; 3 Department of Dermatology, New York University Langone Medical Center, New York, New York, United States of America; 4 Department of Pathology, New York University School of Medicine, New York, New York, United States of America; 5 Center for Neurologic Diseases, Brigham and Women´s Hospital and Harvard Medical School, Boston, Massachusetts, United States of America; Instituto de Medicina Molecular, Portugal

## Abstract

Notch signaling is essential for the development of T cell progenitors through the interaction of NOTCH1 receptor on their surface with the ligand, Delta-like 4 (DLL4), which is expressed by the thymic epithelial cells. Notch signaling is quickly shut down once the cells pass β-selection, and CD4/CD8 double positive (DP) cells are unresponsive to Notch. Over the past two decades a number of papers reported that over-activation of Notch signaling causes T cell acute lymphoblastic leukemia (T-ALL), a cancer that prominently features circulating monoclonal CD4/CD8 double positive T cells in different mouse models. However, the possible outcomes of Notch over-activation at different stages of T cell development are unknown, and the fine timing of Notch signaling that results in T-ALL is poorly understood. Here we report, by using a murine model that ectopically expresses DLL4 on developing T cells, that the T-ALL onset is highly dependent on a sustained Notch activity throughout the DP stage, which induces additional mutations to further boost the signaling. In contrast, a shorter period of Notch activation that terminates at the DP stage causes a polyclonal, non-transmissible lymphoproliferative disorder that is also lethal. These observations resolved the discrepancy of previous papers on DLL4 driven hematological diseases in mice, and show the critical importance of the timing and duration of Notch activity.

## Introduction

The Notch pathway is highly conserved in multicellular eukaryotes and essential in controlling spatial patterning, morphogenesis and homeostasis in embryonic and adult tissues [1,2]. 

The Notch pathway involves five ligands, four NOTCH receptors, and sequential proteolytic processing of the ligand-bound receptors to generate active Notch intracellular domain (NICD), a process in which the proteolytic activity of γ-secretase is crucial [1,3]. We have shown that combined deletion of the two proteolytic subunits of the γ-secretase complex, presenilins 1 and 2 (PS1 and PS2), results in complete ablation of Notch activity in T-cells [4]. Once NICD is generated, a transcriptional program is executed, which includes *Hes* and *Hey* family members, *Deltex-1* or *Myc*, depending on cell type and context [5]. 

The thymus provides a unique environment for T cell development [6]. T cell development starts when an early progenitor enters the thymus and interacts with thymic epithelial cells (TEC) expressing Delta-like ligand 4 (DLL4) [7,8]. When DLL4 is not expressed on TEC, T cell development is abrogated. Enforced expression of NICD in hematopoietic stem cells (HSC) bypassed the elimination of DLL4 in TEC [7]. DLL4 induces T cell commitment exclusively via interaction with the Notch1 receptor [9]. The Notch pathway is essential for T cell development until β selection [10-12] but after that stage it is dispensable [4,12]. 

In mammals, *NOTCH* was discovered as the T cell receptor partner of a chromosomal translocation that resulted in T-ALL [13]. Since then, the Notch pathway has been linked to several types of cancer, and, depending on the tissue, can function as an oncogene [14-17], as a tumor suppressor [18,19], or even have both roles, depending on which Notch receptor is inactivated [20,21]. T-ALL is probably the most studied Notch-mediated cancer [22,23], with NOTCH1-activating mutations found in about 50% of T-ALL patients [24], and 8-12% displaying mutations in FBW7, a molecule involved in the degradation of NICD [25,26] . However, while a hyperactive NOTCH pathway is observed in virtually all T-ALL cases, a subset of patients does not have pathway-activating mutations in NOTCH1 or FBW7, or the *TCR: NOTCH1* translocation; despite extensive analyses, no other mutations in the Notch pathway have been linked to T-ALL in human patients [27,28].

Two laboratories reported the reconstitution of mice with bone marrow cells ectopically expressing DLL4 [29,30]. Surprisingly, the outcomes were very different. While the report of Yan et al, showed a transferable clonal T-ALL in 60% of the recipients, the work by Dorsch et al showed a non-clonal non-transferable lymphoproliferative disease. 

In this report, we present two new mouse models. One is called Tg8, in which DLL4 is ectopically expressed, under the transcriptional control of the TCRα, on the surface of developing and mature T cells beginning at the DP stage. All Tg8 mice succumb to T-ALL at a young age. The second mouse model is Tg8 crossed with Presenilin conditional (floxed) knock-out and CD4-cre mice (Tg8 PS KO CD4-Cre). In this model, Notch signaling is genetically abrogated at the DP stage. These DP cells do not become transformed and T-ALL does not occur. However, due to ectopic Notch signaling on precursors outside the thymus, there is an uncontrolled accumulation of polyclonal DP cells that results in massively enlarged secondary lymphoid organs. These results define an exquisite developmental window for Notch signaling effects, and help explain the discrepancy between the previous reports on DLL4 induced hematological diseases [29,30].

## Materials and Methods

### Ethics Statement

All procedures were approved by New York University’s Institutional and Animal Care Use Committee (IACUC).

### Mice

Tg8 mice were generated as described, using the same MBP-specific *TCRα* construct [31]. 

Tg8 mice and control Tg5 mice were generated with exactly the same *TCRα* DNA preparation, by microinjection into C57BL/6 fertilized eggs.

*PS1* ^fl/fl^ PS2^-/-^ (PS KO) CD4-Cre mice were previously described [4]. Tg8 PS KO CD4-Cre mice were generated by crossing the two aforementioned strains. Nu/nu B6 and B6 mice were purchased from Jackson labs (Bar Harbor, ME). 

### Antibodies and stainings

Thymocytes and splenocytes were stained and run through BD LSRII. Data were analyzed with the FloJo software (Tree Star). Antibody list is provided below. 

### BrdU labeling and Propidium Iodide (PI) staining

 BD Pharmingen BrdU Flow Kits were used for BrdU staining. Mice were injected i.p with 1mg of BrdU in PBS and sacrificed 4 hours later. Single cell suspensions were prepared form thymus, spleen, and mesenteric LN. For PI staining, we followed established procedures [32]. 

### Real time PCR

RNA was extracted using Trizol Reagent (Invitrogen) according to manufacture’s instructions. Total RNA was reverse transcribed into cDNA using SuperScript Reverse Transcriptasae II (Invitrogen). Primers are listed below.

### Notch1 and Fbw7 sequencing

cDNA was synthesized from Tg8 pre-tumoral and tumoral spleens. PCR primer listed below.

### Dll4 knock-down, Bone Marrow infection, T cell transduction

*Dll4* shRNA was ligated into pQXIP-GFP. The same amount of empty vector was transfected as a control. BM cells were cultured in Optimen with SCF, FLT3L, IL6 and IL7 for 24 hrs before infection. Virus supernatants were collected and added to BM cultures. Two days later, 5x10^6^ GFP^+^ cells were injected i.v into RAG1^-/-^ recipients. 

### Antibodies

anti-CD4 (clone H129.19, BD bioscience), anti-CD8 (53-6.7, Biolegend), anti-DLL4 monoclonal antibody (mAb) YM152F: courtesy of Dr. M. Yan, Genentech; final staining concentration: 1 μg/mL, Goat F(ab’)2 anti-human Ig(gamma)-PE as secondary antibody (Invitrogen, H10104), anti-Notch1 (HMN1-12, Biolegend), anti-NICD (mN1A, eBioscience, an antibody that reacts with the intracellular domain of Notch1 but has very low affinity for full-length Notch1) [33]. For intracellular Notch1 staining, cells were fixed and permeabilized using eBioscience Fixation and Permeabilization Kit, followed by incubation with 10μg/ml mN1A at room temperature for 30 minutes. anti-Vβs: Vβ3 (JOVI.1, Caltag), 4(KT4, Caltag), 5.1/5.2 (MR9-4, PharMingen), 6(RR4-7, BD), 7(TR310, Caltag), 8.1/8.2 (MR5-2, BD), 8.3(1B3.3, PharMingen), 9 (MR10-2, BD), 12(KT12, Caltag), 13(mr12-3, BD) and 14(14-2, PharMingen), anti-CD25 (PC61.5, eBioscience), F4/80 (BM8, Biolegend), CD45 (Biolegend #103128; Clone: 30-F11). For immunohistochemistry, the following antibodies were used: anti-CD4 Alexa 647(Biolegend #100531; clone: RM4-5), anti-CD8 Alexa 488 (Biolegend #100723; clone: 53-6.7), anti-CD4 615 (eBioscience #42-0042-80; clone: RM4-5)

### FoxN1 polymorphism PCR

PCR was performed to determine the polymorphism in the *FoxN1* gene. After PCR, the product was digested with Bsaj 1 for 2 hours at 60°C. The sample was analyzed by electrophoresis in a 4% Nusieve agarose gel. When indicated, CD4 cells were purified by MACS using Miltenyi reagents and a Vario MACS apparatus (Miltenyi Biotec, Auburn, CA) following the manufacturers’ instructions.

### Primers

For real-time PCR

Dll4 F: CGCCAGGAAACTCTCTCATC; R: TCATTTTGCTCGTCTGTTCG

Hes*-*1 F: TGTCTGCCTTCTCTAGCTTGG; R: GCGAAGGGCAAGAATAAATG

Hey1 (Hrt-3) F: CACTTGAAGATGCTCCATGC; R: TTCCCGAAACCCAATACTCC

*Deltex-1* F: GAGGTCCACCAGCGTCAG; R: GCCAGTGCCATTCAAGTTCT

*Notch1* F: CATCCGTGGCTCCATTGTCTACC; R: AGGCTCCACCGGCTCACTCTT

*Notch3* F: TTCCCAGCGAGCATCCTTATTTGA R: AGTTGCTGGGCTAGGTGTTGAGTC

For Notch1 and Fbw7 sequencing

Notch1 

Exon 25-29 F: CCTCTTTGATGGCTTCGACT; R: AGCACCATCTGAGGCATTCT. 

Exon 34 3’F: GTGAGCTCGGCAGCCAAT; R: CCAGGAAAATCAAGGCTCTG. 

Exon 34 5’ F: AGCACTGGCTGGAGGTAGC; R: GAGGATGGCAGTGATGTGG. 

Fbw7

Exon 9-10 F:GGCAACCGCATAGTTAGTGG; R: GAGATCCACTCACCACATGG

Exon 12 F: CTGGGAATGCAGATTCTACAG; R: TATTGTCTACACAGTTGGAC

*Ptcra* (*pre-Tα*)a F: AATAGATCTCTACCATCAGGCATCGCT

R: AATCCGCGGCTACTGGAGGTGCTGGCCCGC

Ptcra (pre-Tα)b F: AATAGATCTCTACCATCAGGGGAATCT

R: AATCCGCGGCTACTGGAGGTGCTGGCCCGC

*TCR* Cα F: TGTTCACCGACTTTGACTCC; R: TGGCGTTGGTCTCTTTGAAG

### Statistical Analysis

Every experiment had multiple repeats (>3 times) with at least 3 animals each time. Statistical analysis were conducted with GraphPad Prism software. The Student's unpaired t test was used as statistical test for significance. p values < 0.05 was considered significant. Error bars denote ± SEM.

## Results

### Tg8 mice spontaneously develop T cell lymphomas with early onset and 100% penetrance

**Figure 1:**
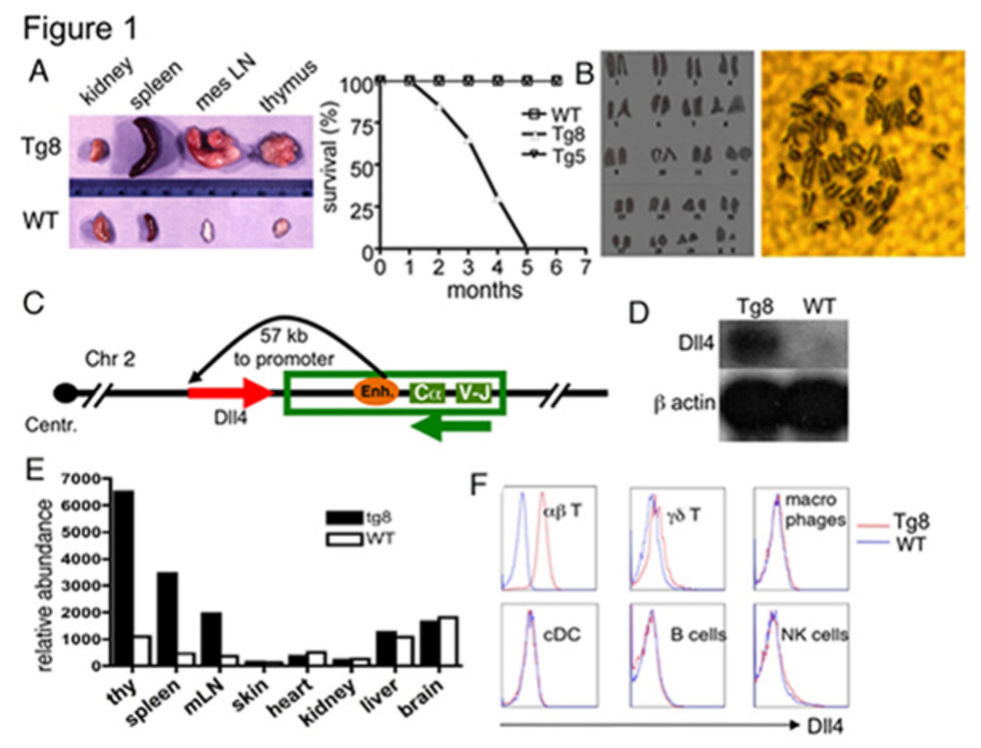
Tg8 mice develop T-ALL due to over-expression of Dll4 gene on T cells. **A**) Enlarged hematopoietic organs (spleen, mesenteric lymph node and thymus) but not non-hematopoietic organ (kidney) of 4 month-old Tg8 mice compared with WT (left panel). Survival curve of Tg8 mice compared to WT and Tg5 mice (right panel). **B**) No obvert aneuploidy in Tg8 lymphomas. On the right panel, a metaphasic spread of one lymphoma; the left panel shows the chromosomes cut from the right side picture and positioned according to their size. Representative of 10 mice. **C**) Position and orientation of *TCRα* transgene and Dll4 gene in mouse Chromosome 2. **D**) Northern blot of Dll4 mRNA expression in Tg8 and WT thymocytes. The same membrane was reblotted with a probe of β actin as a loading control. **E**) qPCR analysis of Dll4 mRNA expression in Tg8 pre-tumoral thymus, spleen, mesenteric lymph nodes (mLN), skin, heart, kidney, liver and brain compared to WT mice. The abundance is calculated as the relative transcription normalized to β-actin expression fixed at 10,000 (left). **F**) DLL4 expression is restricted to αβ T cells. Flow cytometry analysis of DLL4 surface expression co-stained with anti-*αβ* TCR (*αβ* T cells), anti-*γδ* TCR (γδ T cells), anti-F4/80 (macrophages), anti-CD11c (dendritic cells), anti-CD19 (B cells) and anti-NK1.1 (NK cells), obtained from spleens of 4 week-old pre-tumoral Tg8 mice and WT littermates. Representative of 5 mice.

Tg8 mice were generated from the making of a series of myelin basic protein - specific T cell receptor (MBP-TCR) transgenic mice. The MBP-TCRα genomic construct is a 40 kb Va4-Ja48 and contains promoter, enhancer and silencer elements required for tissue specific expression in the T cell lineage. The construct was inserted at random sites of the genome to generate a series of Transgenic (Tg) mouse lines. In one line, which was named Tg8, the construct was inserted on Chromosome 2. We determined that the exact insertion site had 100 percent correlation with probes D2Mit164 and D2Mit423 (MIT/Whitehead Genome Center), located in chromosome 2 at 71 cM and 68.9 cM. Interestingly, we observed spontaneous T cell lymphomas (TCL) in all Tg8 mice (Figure **1A**), but not in mice that have the same TCRα transgene inserted into a different chromosome (Tg5). In Tg8 mice, enlarged inguinal or axillary lymph nodes were palpable at about 3 months of age. Approximately one month after that, all mice died (Figure **1A**). Tg8 lymphomas were clonal, i.e. virtually all T cells of a given mouse expressed the same TCRβ chain, and the selection of the Vβs appeared to be stochastic (Figure **S1A**). TCL were transferrable (see below). All Tg8 TCL co-expressed CD4 and CD8 (CD4^+^CD8^+^ double positive or DP) to different degrees (Figure **S1B**). The high frequency of circulating DP cells at later stages (>>25%) makes Tg8 a spontaneous model of T cell acute lymphoblastic leukemia (T-ALL). At the cytogenetic level, no aneuploidies or translocations were found in Tg8 tumors (Figure **1B**), indicating no major genomic instability. No tumor arose in other tissues. 

Only one copy of the TCRα transgene was inserted in Tg8 DNA (Figure **S1C**, **left and central**). The size of the *TCRα* transcript was not altered by the insertion (Figure **S1C**, **right**). 

### Ectopic expression of Delta-like ligand 4 (DLL4) on the surface of Tg8 T cells

The *TCRα* genomic construct used in the making of Tg8 mice contains promoter, enhancer and silencer elements required for tissue specific expression in the T cell lineage [34-37] (Figure **1C** and Figure **S1D**). These transcriptional regulatory elements could regulate the expression of the genes surrounding the insertion site in Tg8 T cells. We therefore cloned and sequenced the transgene insertion site and assessed transgene-associated changes in expression of all neighboring genes (Figure **S1E** and **S1F**). *Dll4* mRNA was elevated about 100-fold in T cells (but not B cells) from Tg8 mice, higher than the positive control of *Dll4* expression, vascular endothelial cells (Figure **S1F**). The overexpression of *Dll4* was confirmed by northern blot, which also indicated that *Dll4* mRNA size was unchanged in Tg8 mice. (Figure **1D**). Only the lymphoid organs expressed high level of *Dll4* (Figure **1E**). Surface staining with anti-DLL4 antibodies showed that αβ T cells expressed DLL4, but not γδ T cells, dendritic cells, macrophages, natural killer cells or B cells (Figure **1F**).

The expression of none of the other neighboring genes was altered in a way comparable to *Dll4* (Figure **S1E** and **S1F**). *Chac1* expression was upregulated in Tg8 T cells, but still remained at a very low level, well below that of *Chac1*-expressing skin Langenhans cells (Figure **S1F**)**.**

*Lmo2* gene is located more than 10 Mb form the Tg8 transgene insertion site on mouse Chromosome 2. Nevertheless, we tested expression levels of *Lmo2* due to its relevance in lymphomagenesis [38], and observed that *Lmo2* expression was not affected (Figure **S1E**). 

**Figure 2:**
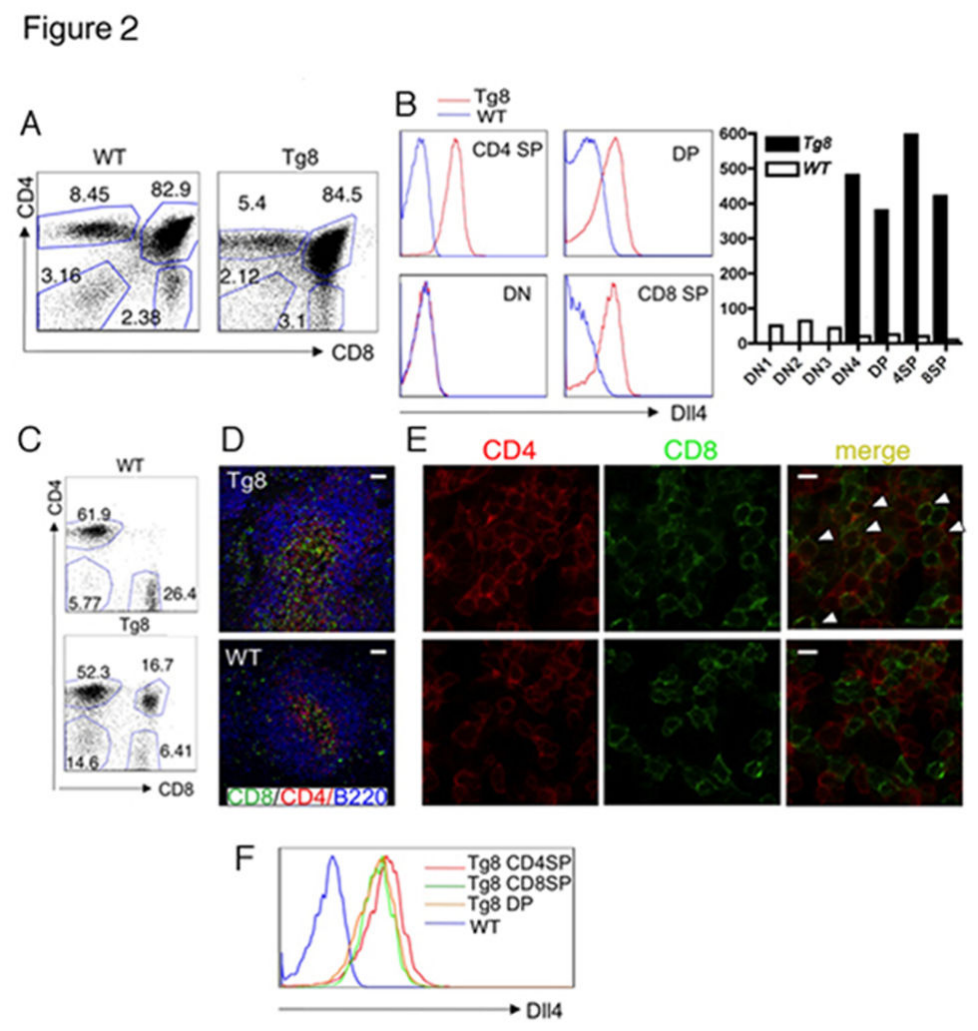
Characterization of Tg8 cells. **A**) Pre-tumoral Tg8 and WT thymus have similar distribution of the major lymphocyte populations (DN, DP, CD4 SP and CD8 SP). Representative of more than 20 mice. **B**) Surface DLL4 staining of the major thymic populations shown in Figure 1F (left) and mRNA level of Dll4 in DN, DP and SP thymocytes calculated by real-time PCR (right). **C**) Pre-tumoral spleens from Tg8 mice accumulate CD4^+^CD8^+^ DP cells. Shown are representative spleens from 4 week-old Tg8 and WT mice. **D**) Normal splenic architecture in pre-tumoral 3 week-old Tg8 mice. Low power resolution confocal images of Tg8 (top) and WT (bottom) frozen spleen sections. The white pulp can be identified by T cell staining (anti-CD4 and anti-CD8) and B cell staining (anti-B220), and the surrounding red pulp appears in dark. White bar = 100 μm. **E**) High power resolution of the PALS area of the spleen. Arrowheads point to some of the DP cells. White bar = 6.25μm. **F**) Peripheral T cells in Tg8 mice, but not WT mice, express surface DLL4. CD4 SP, CD8 SP and DP cells, gated as in Figure 2C), were stained with anti-DLL4. Representative of more than 20 mice.

In the thymus of pre-tumoral Tg8 mice, the main thymic subpopulations had a relatively normal distribution (Figure **2A**). DLL4 surface expression was negative in CD4^-^CD8^-^ double-negative (DN) cells, began at the DP stage and was maintained in both CD4 single positive (SP) and CD8 SP cells (Figure **2B** **, left**). Preceding surface expression, *Dll4* mRNA was expressed at the DN4 stage (Figure **2B** **, right**). In pre-tumoral spleens, red pulp and white pulp zones were readily found, and the white pulp retained well-defined T and B cell zones (Figure **2D**). Interestingly, from a young age, much before the tumoral stage, there were CD4^+^CD8^+^ (DP) cells in circulating blood and spleens of Tg8 animals, as assessed by flow cytometry (Figure **2C**) and immunohistology (Figure **2D and 2E**). The percentage of B cells was normal (Figure **S1G**). In the periphery, CD4 SP, CD8 SP and DP cells all expressed DLL4 on their surfaces (Figure **2F**). 

### The Notch pathway is activated in DP cells of Tg8 mice

**Figure 3:**
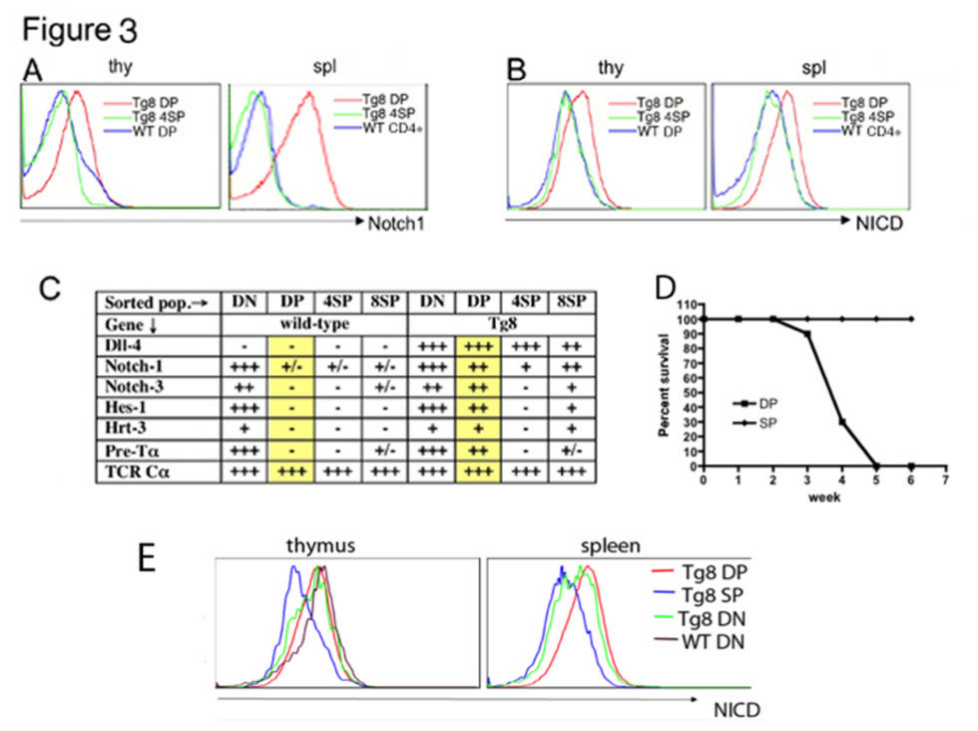
The Notch pathway is upregulated in DP cells of Tg8 mice. **A**) Notch1 receptor surface staining of Tg8 DP, CD4 SP and WT DP in thymus (thy) or WT CD4^+^ T cells in spleen (spl). **B**) Notch intracellular domain (NICD) staining of Tg8 DP, CD4 SP and WT DP in thymus (thy) or WT CD4+ T cells in spleen (spl). **C**) Thymic DN, DP and SP populations from Tg8 mice and WT littermates were sorted and cDNA was prepared for real time PCR analysis of the indicated genes. Summary of quantitative real time PCR data from three independent sorting experiments. Expression: - <5; +/- 5-15; + 15-50; ++ 50-100; +++ >100, normalized on β-actin expression fixed at 10,000. The yellow shade highlights the DP populations, which display the largest differences between WT and Tg8 mice. **D**) 5x10^6^ DP or SP cells were sorted from the same Tg8 mice at the early tumorigenic stage, and injected to nude mice. Survival rates of the recipients were recorded in the following 5 weeks. **E**) NICD staining of Tg8 DN, DP, SP and WT DN cells in thymus and spleen.

Given the over-expression of DLL4 on T cells, we investigated the state of the Notch pathway in Tg8 mice. Among the four Notch receptors, NOTCH1 plays a non-redundant role in T cell development[9,39]. DP cells from thymus and spleen of Tg8 mice had markedly higher surface NOTCH1 expression than DP cells from WT thymus. Interestingly, surface levels of NOTCH1 in Tg8 CD4 SP were very similar to their WT counterparts, indicating a down-regulation after the DP stage (Figure **3A**). Next, we stained for the activated form of NOTCH1, NICD. Tg8 DP cells had higher NICD levels than Tg8 CD4 SP cells and WT CD4^+^ cells (Figure **3B**). qPCR analysis of the expression of several Notch target genes in purified DN, DP, CD4SP and CD8SP cells showed that, in WT T cells, expression of *Hes1*, *Heyl* (*Hrt3*), and *Ptcra* (*Pre-Tα*) undergoes a profound reduction as cells transition from the DN to the DP stage. However, in Tg8 cells, expression of Notch pathway target genes remained high at the DP stage (Figure **3C**). The expression of Notch target genes was downmodulated in Tg8 SP cells, in agreement with the downmodulation of the NOTCH1 receptor at the same stage (Figure **3C**). There is very good correlation between surface NOTCH1 expression and its mRNA level in the different T cell populations (Figure **3A and 3C**) [40].. In order to test if DP cells and/or SP cells were transformed malignant tumor cells, we injected equal numbers of DP cells or SP cells at an early tumoral step from the same Tg8 mice to the immuno-compromised recipients. As shown in Figure **3D**, all the animals injected with Tg8 DP cells succumbed to T-ALL five weeks after cell transfer, while the animals injected with SP cells didn’t develop cancer. This indicates that Tg8 DP cells, but not SP cells, are transformed malignant cells. Tg8 DN cells had high NICD levels similar to Tg8 DP cells and WT thymic DN cells (Figure **3E**), indicating that DN cells in both Tg8 and WT receive DLL4 signals and have activated Notch pathways. 

We also determined the surface expression level of CD25, another Notch target gene. In accordance to the NICD staining and qPCR analysis of other Notch target genes, CD25 expression was elevated in Tg8 DP cells, but low in Tg8 CD4SP cells and WT cells (Figure **S2A**). All these results clearly demonstrate that the Notch pathway is hyperactive at DP stage of pre-tumoral Tg8.

In order to determine if the hyperactive Notch pathway led to a higher cell division rate at the pre-tumoral stage, we measured BrdU incorporation and carried out cell cycle analysis with propidium iodide staining. We found no differences between Tg8 and WT T cells, either in thymus, spleen or LNs (Figure **S2B** and **S2C**) demonstrating that the active Notch pathway did not promote cell cycle entry at the pre-tumoral stage. 

### Blockade of the Notch pathway in Tg8 mice impairs lymphoma development

**Figure 4:**
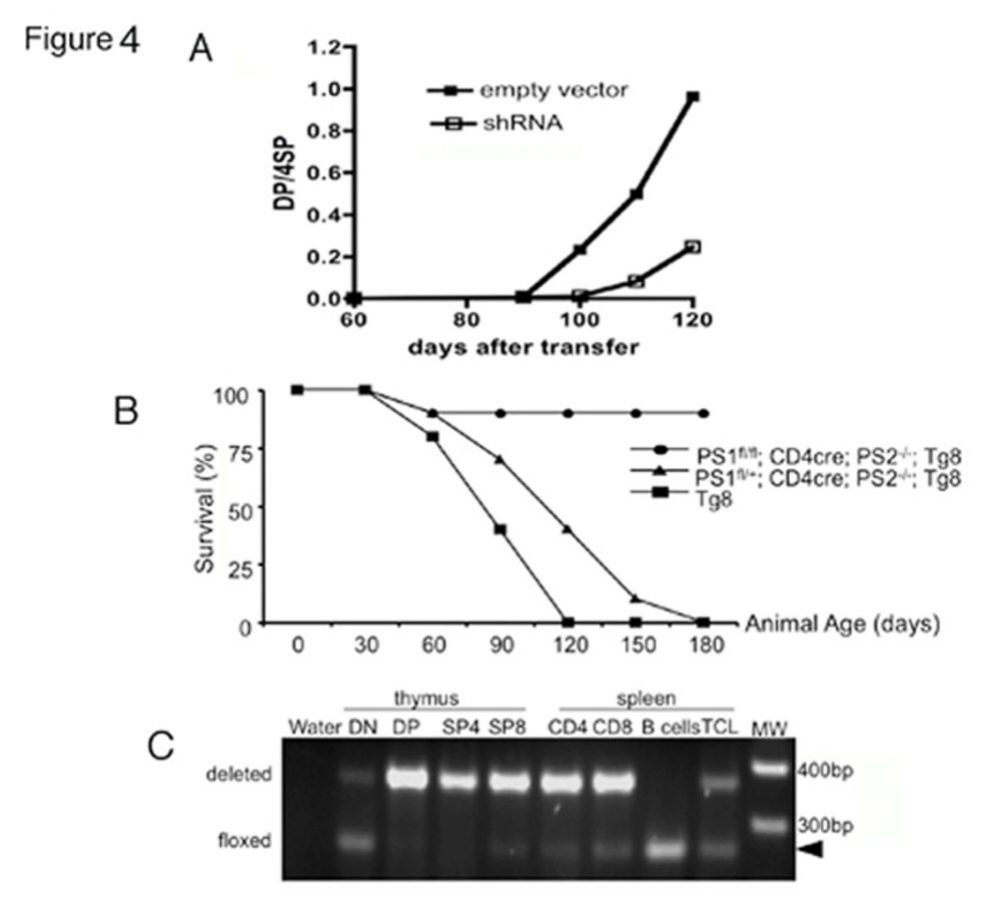
blockade of Notch signaling rescued Tg8 from developing T-ALL. **A**) Dll4 shRNA delays the onset of Tg8 lymphoma. The appearance of DP cells was monitored by blood staining every 10 days. Data are presented as the change in the DP/CD4 SP ratio over time. n=3 mice per group. **B**) Conditional KO of γ-secretase in T cells eliminates Tg8 lymphomas. Survival curve of Tg8 PS KO (PS1^fl/fl^ PS2^-/-^) CD4-Cre mice (or PS1^fl/+^ as control) (n=10 animals per group). The survival curve of 10 Tg8 mice was also recorded. Note that one PS1^fl/fl^ mouse escaped complete PS1 deletion (as shown in panel C) and developed lymphoma. **C**) Efficiency of CD4-Cre-mediated PS1 deletion in different purified thymic and splenic cell populations. TCL: DNA from the only T cell lymphoma observed in the Tg8 PS KO CD4-Cre group. Arrowhead: non-deleted PS1 allele (Floxed).

To evaluate if eliminating the Notch pathway in Tg8 T cells would stop T-ALL, we infected Tg8 hematopoietic stem cells (HSC) with a retroviral construct harboring a short hairpin (sh) against *Dll4* (sh*Dll4*), and reconstituted irradiated RAG1^-/-^ mice with HSC infected with either sh*Dll4* or the empty vector. Since pre-tumoral Tg8 mice gradually accumulate DP cells in the blood, we analyzed the percentage of DP cells among the CD4^+^ T cells in blood every 10 days as a way to measure progression of the disease. There was a clear delay in the appearance of DP cells in mice reconstituted with sh*Dll4*-infected HSC compared to those with empty vector-infected HSC (Figure **4A**), indicating that the rise of DP cells in Tg8 mice was driven by DLL4. However, DP cells were ultimately observed in the blood of these mice, due to the fact that sh*Dll4* reduced DLL4 expression only partially and temporarily (Figure **S2D** and **S2E**). Four months after sh*Dll4*-HSC injection the effect of the sh*Dll4* had been totally lost (Figure **S2E**). The temporal nature of the effect of the shRNA meant that a different genetic approach was needed to permanently abolish Notch signaling. We have previously shown that inactivation of both PS1 and PS2 in T cells, using CD4-Cre *PS1* ^fl/fl^ PS2^-/-^ mice (PS KO CD4-Cre) [41], abrogated Notch signaling [4]. We therefore crossed Tg8 mice with PS KO CD4-Cre mice. Strikingly, these mice became resistant to TCL development. From a group of 10 Tg8 PS KO CD4-Cre mice, only one mouse developed TCL (Figure **4B**), and this was the only animal that retained a functional *PS1* ^fl^ allele (Figure **4C**, lane labeled TCL, non-deleted *PS1* allele indicated by the arrowhead). In contrast to Tg8 PS KO CD4-Cre mice, 19 of 19 Tg8 littermates that either lacked CD4-Cre or were *PS1* ^fl/+^ developed TCL. Interestingly, Tg8 CD4-Cre *PS1* ^fl/+^ PS2^-/-^ mice showed a slight delay in tumor onset compared to Tg8 CD4-Cre PS1^+/+^ PS2^-/-^ mice, probably due to lower Notch activation (Figure **4B**). 

Taken together, these results demonstrate that expression of a functional *Dll4* gene on the surface of T cells leads to Notch pathway activation, causing T-ALL. 

### Swift transition from polyclonality to monoclonality in Tg8 mice indicates secondary tumorigenic events

We took advantage of the complete penetrance of T-ALL in Tg8 mice to study the pre-tumoral events. First, we determined that the DP cells circulating in young Tg8 mice were polyclonal, while the TCL were always clonal. Thus, at some point a DP clone gains the advantage over the remaining DP cells. We therefore studied the polyclonal to clonal (i.e. malignant) transition in 13 Tg8 mice, all of which developed TCL. 

**Figure 5:**
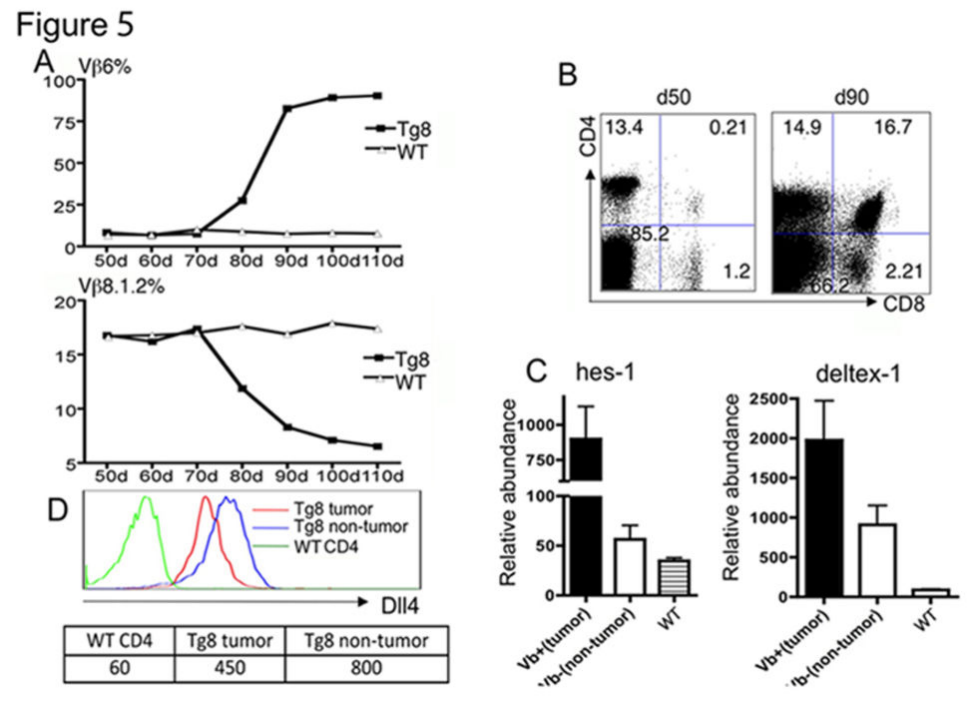
Swift transition from polyclonality to monoclonality in Tg8 mice is associated with further upregulated Notch. **A**) Blood from Tg8 and WT mice was drawn every 10 days starting at age 50d and stained with different TCR Vβ antibodies. Shown are DP-gated T cells from a case in which a Vβ6^+^ (top panel) TCL arose, with resulting decrease in the proportion of Vβ 8.1.2 (bottom panel) T cells. The Vβ usage in WT mice was calculated based on CD4^+^ T cells. **B**) Accumulation of DP cells over time in the blood shown in panel A). **C**) Clonality is associated with increased Notch signaling. Thirteen Tg8 mice were followed as in Figure 3A with a panel of Vβ antibodies: Vβ 3, 4, 5.1.2, 6, 7, 8,1,2, 8,3, 9, 12, 13 and 14. In six of these mice, the early clonal burst could be identified by one of the listed Vβ antibodies. In these 6 mice, clonal DP cells (Vβ ^+^) and remaining polyclonal DP cells from the same mice (Vβ^-^) were sorted. Expression of *Hes-1* and *Deltex-1* was determined by qPCR. Shown is mean +/- SEM. **D**) Tg8 tumor cells do not further up-regulate DLL4 expression. Flow cytometry was carried out as in Figure 1. Mean Fluorescence Intensity (MFI) was shown at the bottom.

By Vβ staining (6 of 13 TCL) and Vβ PCR (the remaining 7 TCL), we observed that all the Tg8 tumors expressed a single Vβ (Figure **5A** and data not shown). Figure **5A** shows an example of the rise of a Vβ6 clonal tumor, with concomitant decrease of the frequency of Vβ8.1.2 T cells. The percentage of DP cells in this mouse increased dramatically between day 50 and day 90, and the bulk of the increase could be attributed to the rise of the clonal Vβ6 DP cells (Figure **5B**).

To study the changes that accompany the transition to malignancy in Tg8 T cells, we compared the rising clonal DP cells with the remaining polyclonal DP cells from the same animals; we chose day 90, an age in which both cell populations could be sorted. We used the spleens of the 6 mice mentioned above with Vβ usage identifiable by antibodies to study the expression of Notch target genes. In all cases, the clonal DP cells expressed higher levels of *Hes-1* and *Deltex-1* than the polyclonal DP cells in the same mice (Figure **5C**), while DLL4 expression levels were not augmented (Figure **5D**). This confirms that the advent of malignancy is associated with a further increase in Notch pathway activation in Tg8 mice. 

One possible explanation for the increase in Notch activity in Tg8 TCL would be the NOTCH1 or FBW7 mutations. We therefore sequenced the *Notch1* and *Fbw7* genes from 18 Tg8 TCL. None of the 18 had any of the known mutations (Figure **S3**). However, four TCL had mutations, in all cases located in *Notch1* exon 34, which encodes the TAD and PEST domain. Unlike the reported T-ALL-associated mutations, none of the 4 mutations resulted in truncation of the PEST domain. 

The sharp transition from polyclonality to monoclonality that we observed in Tg8 mice strongly suggests the existence of secondary tumorigenic events, which further activate Notch signaling. These secondary events are different from the known Notch coding region mutations. 

### Presenilin-deficient Tg8 mice develop a non-malignant lymphoproliferative disease

**Figure 6:**
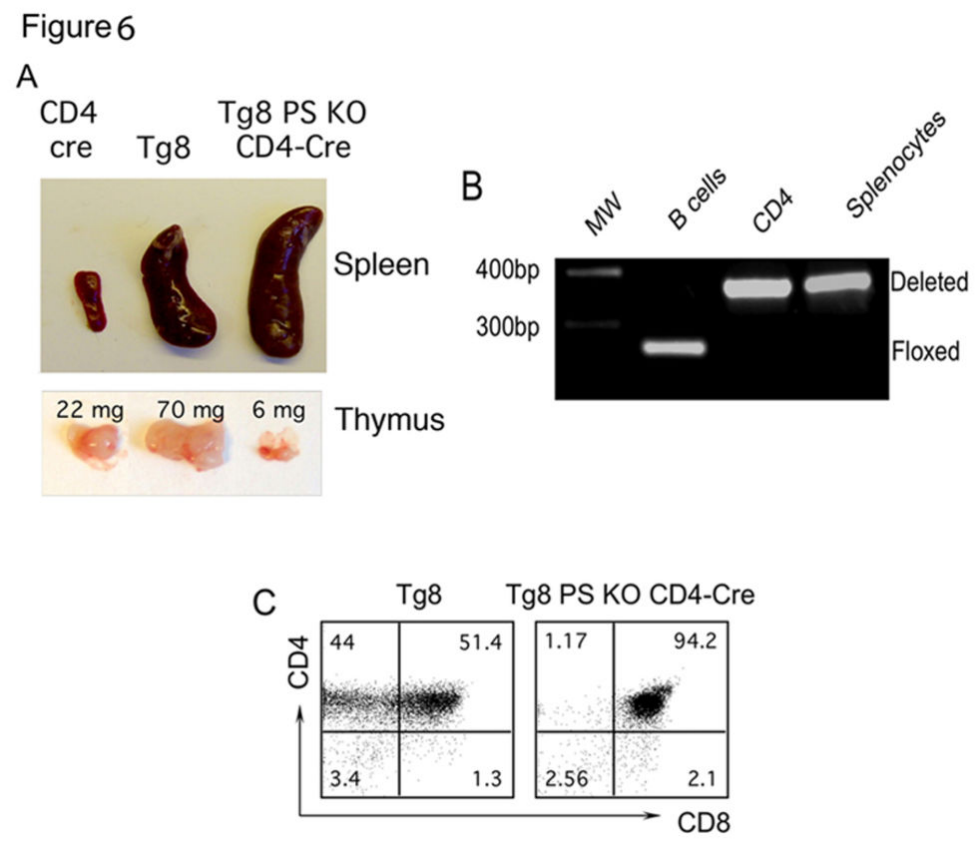
Presenilin-deficient Tg8 mice develop a lymphoproliferative disease. **A**) Size and weight of representative lymphoid organs obtained from 4 month-old Tg8 and 8 month-old Tg8 PS KO CD4-Cre and CD4-Cre mice. Note the small size of the thymus in Tg8 PS KO CD4-Cre as compared to Tg8 mice (lower panel). **B**) Complete Cre-mediated deletion of PS1 in splenocytes from 4 month-old Tg8 PS KO CD4-Cre animals, as determined by PCR Analysis. **C**) Expression of CD4 and CD8 in Tg8 or Tg8 PS KO CD4-Cre splenocytes was examined the same time as 6A).

All Tg8 mice die of T-ALL by the age of 5 months with an average of 4 months. As Tg8 PS KO CD4-Cre mice aged to more than 7 months, they started to display enlarged LNs. These animals ultimately developed a fatal lymphoproliferative disease, with noticeable splenomegaly and enlarged LNs. The thymi of the same mice were slightly smaller than that of their control littermates (Figure **6A**). It was possible that the lymphoproliferative disease in Tg8 PS KO CD4-Cre was a delayed T-ALL caused by incomplete deletion of the floxed PS1 alleles. However, this was not the case, as we did not observe the floxed *PS1* band (Figure **6B**).

**Figure 7:**
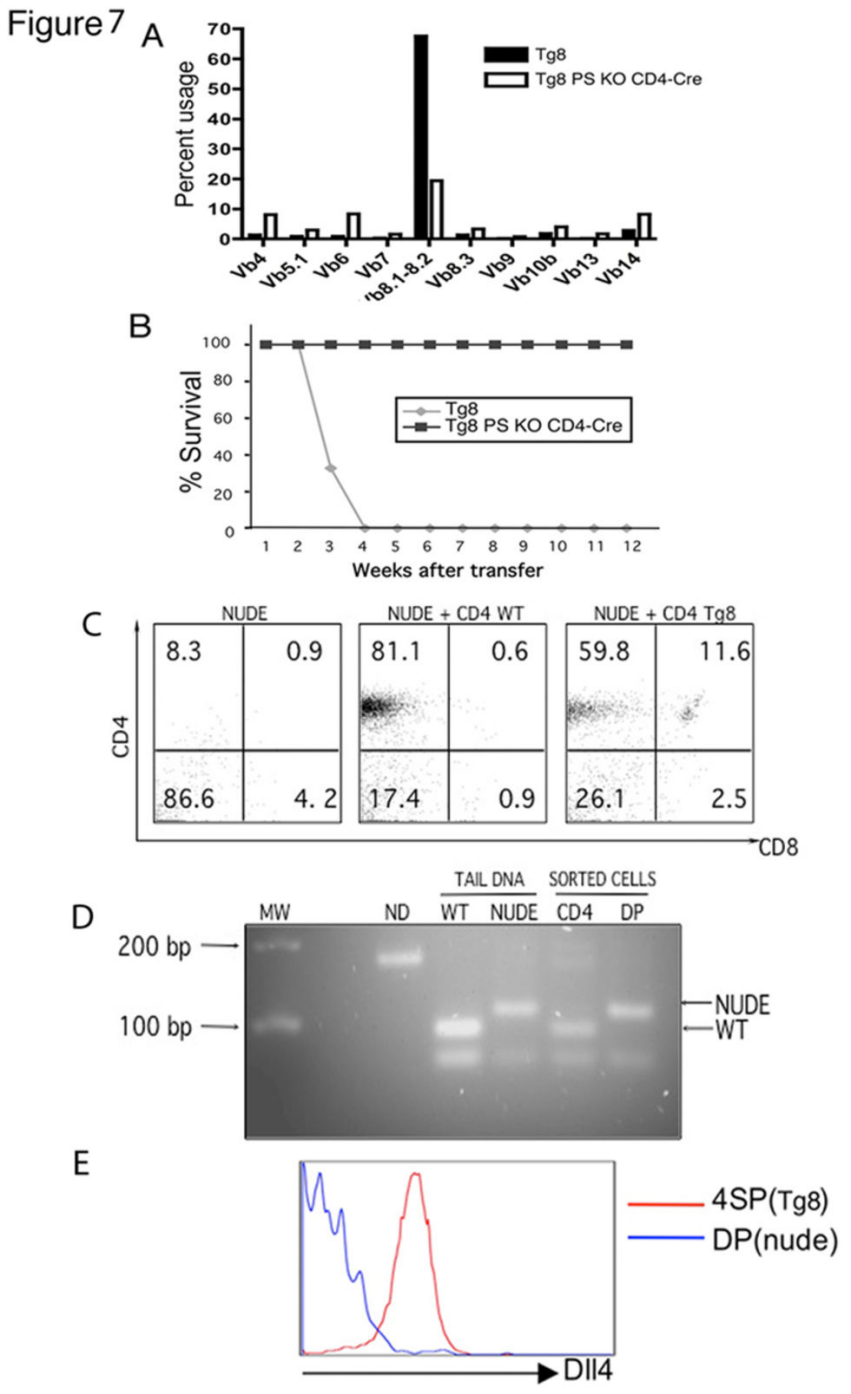
the lymphoproliferation in Tg8 PS KO CD4-cre is non-clonal and non-transferable. **A**) Mice harboring T cells deficient in γ-secretase retain a polyclonal T cell repertoire. Vβ antibody staining of gated CD4^+^ T cells of 8 month-old Tg8 PS KO CD4-Cre and a Tg8 mouse of 4 months with a Vβ8.2^+^ lymphoma. **B**) Tg8 PS KO CD4-Cre T cells from A) do not transfer malignancy, in contrast to the transfer of malignancy by Tg8 splenic T cells from A), as shown in the survival curve of nude recipient animals that were injected with 5x10^6^ CD4^+^ splenocytes from Tg8 or Tg8 PS KO CD4-Cre mice. **C**) CD4 and CD8 staining profile of CD3^+^ splenocytes from nude animals that were injected one month before with PBS (PBS), WT CD4^+^ cells (CD4 WT) or Tg8 CD4 SP T cells (CD4 Tg8). **D**) The origin of the different splenocyte populations in Figure **7C** was determined by *Fox* *N1* gene profile. Tail DNA as template was used for amplification and digestion of the control bands for both WT and nude (NUDE). The non-digested PCR product (without date) is also shown. MW: molecular weight markers. **E**) Surface staining of DLL4 on CD4 SP and DP cells further supports the nude origin (DLL4-negative) of DP cells.

We analyzed the cells that accumulated in 8-month old moribund Tg8 PS KO CD4-Cre animals. Virtually all cells in the spleen and the LNs of these animals were DP (Figure **6C**). The analysis of the Vβrepertoire showed that they were polyclonal, while DP cells in Tg8 TCL were clonal (Figure **7A** shows an example of a Vβ8.1.2^+^ Tg8 TCL). This result suggested that the lymphoproliferative disease in Tg8 PS KO CD4-Cre mice was not cancer, but another form of lymphoproliferation. To test this hypothesis, we transferred DP cells from Tg8 TCL (at 4 months of age) or Tg8 PS KO CD4-Cre (at 8 months of age) mice into nude recipients. After 3 to 4 weeks, all recipients of Tg8 TCL died of T-ALL (Figure **7B**). In contrast, the animals that received Tg8 PS KO CD4-Cre DP cells remained free of disease (Figure **7B**), which confirmed that these polyclonal DP cells were not transformed, malignant cells. Although these polyclonal DP cells express DLL4, they have no Notch signaling, and are not sustained as a population without continuous overproduction from Notch-signaling competent (pre-CD4) T cell precursors. Thus, two different types of lymphoproliferative disease are observed in mice that express DLL4 on T cells - a clonal, transferrable malignant disease and a non clonal lymphoproliferative disease. Each of the two outcomes is perfectly predictable based on the developmental window in which the T cells are allowed to receive Notch receptor-mediated signals.

### CD4^+^ T cells from Tg8 mice support the extra-thymic DP development of precursors

DLL4 is very important in T-development both in vitro and in vivo [7]. We therefore reasoned that mature Tg8 cells, which express DLL4 on their surface, drove T cell development in an uncontrolled way. To demonstrate this, we transferred purified CD4^+^ SP cells from pre-tumoral Tg8 or WT mice into nude recipients [42]. Four weeks later, the spleens of nude mice that received WT CD4^+^ T cells had CD4 SP cells only, and they were of donor origin. In contrast, spleens of nude mice that received CD4 SP cells from Tg8 animals displayed a clear DP population (Figure **7C**). The DP cells in nude recipients of Tg8 CD4 SP cells could be of Tg8 or nude origin. By polymorphism in the *Fox N1* gene we showed that DP cells were of nude origin, while CD4 SP cells were of donor Tg8 origin (Figure **7D**). We confirmed these results by DLL4 surface staining, which showed that the CD4 SP cells expressed DLL4 (i.e. Tg8 origin) while the DP cells did not (i.e. nude origin) (Figure **7E**). Our results demonstrate that DLL4-expressing Tg8 T cells can drive the T cell development in trans in vivo. 

## Discussion

Notch was discovered in mammals as a consequence of its intracellular domain NICD being driven by the TCRβ transcription regulatory elements, causing T-ALL in humans [13]. Here we show that the Notch ligand DLL4 can cause T-ALL when placed under the transcriptional control of the *TCRα*, as occurred in Tg8 mice. 

In the present work we report a mouse model showing that Notch signaling on T cells can trigger two different types of diseases. One disease is T-ALL, a clonal, acute (as the name indicates), transferrable disease. The second disease is a non-clonal, non-transferrable lymphoproliferative disease with a much slower progression than T-ALL, but ultimately also lethal. 

T-ALL was the unavoidable outcome of Tg8 mice, a consequence of continuous Notch signaling induced by DLL4 expression on all T cells, while in WT thymus DLL4 expression is restricted to cortical epithelial cells (cTEC). Our results strongly support the notion that the same DLL4-triggered Notch pathway, which is essential for T cell generation at the DN stage, is terribly harmful to the host if not shut down at the DP stage as we forced in the Tg8 PSKO CD4-cre. Tg8 mice display elevated NOTCH1 surface expression and its activated form (NICD) only in DP cells, and, accordingly, DP cells displayed the highest expression of Notch target genes. Compared to the corresponding T cell populations in WT mice, Tg8 mice display elevated expression of surface NOTCH1 and the activated form NICD only in DP cells, and, accordingly, DP cells displayed the highest expression of Notch target genes. In addition, Tg8 DP cells were able to transfer the disease when injected in nude mice while SP cells were not. Taken together, out data suggest that DP cells are the ones at the origin of T-ALL of Tg8 mice.

T-ALL in humans and experimental animals has been correlated with increased Notch signaling, and T-ALL in Tg8 mice is no exception to this rule. There are however, new features revealed by the Tg8 T-ALL model. First, the expression of the Notch ligand DLL4 on T cells, by itself, is sufficient to drive T-ALL in all mice with a short pre-tumoral latency. In no case previously known was DLL4 expression restricted to T cells. Instead, others used Dll4-transduced bone marrow cells [29,30]. Second, we did not find any of the mutations in *Notch1* or *Fbw7* that have been previously associated with 50-60% of human T-ALL cases. However, 3 out 18 Tg8 lymphomas could be accounted for by mutations in PEST domain Ser residues that are associated with stabilization of NICD [43]. It remains the case that, overwhelmingly, Tg8 TCL does not display the known T-ALL mutations in the coding region. Interestingly, with no exceptions, the malignant TCL further up-regulated Notch signaling, indicating that secondary tumorigenic events took place in single pre-tumoral cells that resulted in super-increased Notch signaling and growth advantage over the remaining pretumoral cells. 

The polyclonal lymphoproliferative disease only occurred in Tg8 PS KO CD4-Cre mice in which Notch signaling on T cells was abrogated at the DP stage due to the CD4-Cre-mediated deletion of *PS1* (on a *PS2* KO background). Lack of Notch signaling at the DP stage eliminated malignant transformation. In these Tg8 PS KO CD4-Cre mice, DP T cells were constantly being produced due to DLL4-triggered Notch signaling of DN cells, but, without Notch signaling on DP cells, there was no transformation to a clonal, malignant state. These observations have an implication on regular feed back mechanisms to control T cell generation. In normal situations, T cell development up to the DP stage is controlled by the limited expression of DLL4 by cTEC, but in Tg8 and Tg8 PS KO CD4-Cre mice, there is abundant DLL4 present on the surface of all T cells. Our results in nude animals, where Tg8 CD4 SP cells but not WT CD4 cells promoted the development of DP cells of nude origin clearly demonstrates that this DLL4-induced pathway does not require a thymus, and that T cells expressing DLL4 are highly competent to induce T cell development in vivo up to the DP stage. 

Human T-ALL has a more complex pattern with DN-like and SP-like tumors observed and Notch1 mutations at different T-cell maturation stages. In our model, DN cells are similar in WT and Tg8 mice in regard to Notch signaling, as seen by Hes1 and Hrt3 expression (Figure 3C). However, in addition to the physiologic thymic epithelial expression of DLL4, in Tg8 mice and Tg8 PS KO mice, there is additional DLL4-mediated triggering of Notch signaling, which is provided by the Tg8 DP and SP T cells themselves (DN do not express DLL4 on their surface but receive DLL4 signals). This excess signaling actively promotes differentiation of the DN cells to DP without malignancy, because DN cells are “used to” dealing with Notch, and Notch is required for their differentiation. Malignancy only arises when Notch signaling is un-physiologically carried on into the DP stage, a stage in which Notch is normally shut down, and then secondary mutations begin to occur and accumulate. The former, the enhanced differentiation from DN to DP, occurs in both Tg8 and Tg8 PS KO, but the latter, the accumulation of secondary mutations and malignancy, only occurs when Notch signaling is allowed to persist in the DP stage, as in Tg8 mice but not Tg8 PS KO mice.

Our data helps explain the conflicting reports using bone marrow cells retrovirally transduced with *Dll4* [29,30]. While Yan et al. reported T-ALL development, Dorsch et al. could not obtain a single animal with T-ALL, although all the reconstituted *Dll4*-expressing animals had a non-clonal, non-transferrable lymphoproliferative disease. As retroviral expression was differentially lost, *Dll4* expression may have been shut down earlier in the case of the Dorsch et al study, and be more persistent in the T cell lineage in the Yan et al study. 

Finally, our findings in Tg8 mice also offer a mechanism for the phenomenon of donor cell leukemia. In these cases, following allogeneic hematopoietic stem cell transplantation, the host environment induces leukemia in healthy donor cells. Depending on the study, it is estimated that between 0.1% and 5% of the post-transplant leukemia relapses in humans are caused by donor cell leukemia [44-46]. Since we showed that DLL4-expressing T cells from Tg8 mice could cause T cell development in nude animals, it is tempting to speculate that, in the Tg8 mouse model, a situation similar to donor cell leukemia could be taking place. While ectopic Notch ligand expression by host cells has not been looked at in correlation with human donor cell T-ALL, our studies suggest that Notch ligands may be good candidates to explain, in part, this intriguing clinical phenomenon.

## Supporting Information

Figure S1 **The cellular and genetic characterization of Tg8 tumors.** **A**) Tg8 mice accumulate clonal DP cells. Tg8 tumors are clonal as indicated by dominant usage of one Vβ in each lymphoma (shown are 3 different 4 month-old Tg8 mice (#1, #3, #5, left) comparing to WT littermates (#2, #4, #6, right). **B**) More than 95% of tumors were CD4^+^CD8^+^DP but a small percentage of tumors can display different CD4 and CD8 expression patterns (shown are 6 different Tg8 individuals with lymphomas numbered #7 to #12). **C**) Tg8 mice have inserted a single copy of the *TCR*α transgene, and the *TCRα* is not part of any aberrant transcript. Left panel: Southern blot of DNA extracted from Tg8, WT or Tg5 animals. The probes used are indicated in Figure S1E. The arrows show the 5’ and 3’ flanking sequences in Tg8. Right panel: northern blot probed with a Cα probe and re-probed with β-actin. **D**) Diagram depicting (to scale) the TCRα construct used to generate lymphoma-prone Tg8 and lymphoma-free Tg5 mice. The position of the main enhancer is indicated by a red box. The position of the 5’ and 3’ probes used in Figure S1D is indicated by blue rectangles below the map. **E**) Summary of the transcriptional effects of Tg8 insertion at Chromosome 2 for genes located between 100 and 250 kb on either side of the integration site. The thick line between Exd1 and *Cbp22* indicates the area closest to the integration site (100 kb on each side). qPCR of the indicated genes was performed, and the ratios of relative transcription level of Tg8 tissue and WT tissue were calculated as listed in the right three columns as thymus, spleen and T lymphoma (TCL). The second and third columns to the left indicate whether the raw expression values of the samples are hardly detectable above the negative control (-), slightly above the negative control (+/-), or clearly above the negative control (+). All changes in expression ratios are non-significant. **F**) Transcriptional effects of Tg8 insertion on the genes located nearest the integration site (100 kb on each side). It is noteworthy that *Ino80* alone occupies more than half of this region, and its promoter lies 134 kb away from the TCRα enhancer in Tg8 mice. qPCR analysis was performed on cDNA obtained from sorted T cells, sorted B cells, sorted brain endothelial cells and Langerhans cells (LC cDNA from C57BL6 background as Tg8, a gift from Dr. Miriam Merad). For T and B cells, data are presented as the ratio between the transcription in Tg8 and WT cells. For endothelial cells and Langerhans cells, the ratio is taken between these cells and WT T cells. Data are represented as mean +/- SEM. **G**) Normal frequency of B cells in spleen of 4 w.o pre-tumoral Tg8 mice analyzed by flow cytometry.
(TIF)

Figure S2 **Proliferation profile of Tg8 T cells and the efficiency of knock-down experiments.** **A**) In Tg8 mice, the Notch pathway is preferentially activated in DP cells. Shown is the surface expression of the Notch target gene CD25 in the indicated populations (color-coded). **B**) Four week-old Tg8 and littermate controls were injected intraperitoneally with BrdU and sacrificed 4 hours later. Thymic, splenic, and mesenteric lymph node cells were surface-stained with anti-CD4 and anti-CD8 antibodies, followed by intracellular staining with anti-BrdU antibody. The panel shows the percentage of BrdU^+^ cells among the populations indicated in the x axis. Data are represented as mean +/- SEM. **C**) Propidium iodide staining of permeabilized thymic and lymph node cells from 3 week-old Tg8 and WT littermate (n=3). **D**) CD4 SP splenocytes sorted from 4 week-old Tg8 were cultured and transduced with either *Dll4* shRNA-pQXIP/GFP vector or the same amount of empty pQXIP/GFP vector. On day 3 the cells were analyzed for DLL4 surface expression with two gates on GFP^+^ and GFP^-^ populations. MFI= 12.9 in GFP^-^ and 28.9 in GFP^+^ **E**) 4 week old Tg8 BMs were transduced with either *Dll4* shRNA or empty vector, and transferred into RAG1^-/-^ recipients. CD4 SP cells from blood were analyzed for DLL4 expression at 3 and 4 months after BM transfer. Tg8 BM transduced with empty vector was also transferred into RAG1^-/-^ mice as positive controls for lymphoma development; WT BM was transferred into RAG1^-/-^ mice as a negative control for lymphoma development. MFI= 15.3 (empty vector), 12.6 (shRNA after 4 months), 7.3 (shRNA after 3 months), 1.7 (WT).
(TIF)

Figure S3 **None of the known T-ALL mutations of *Notch1* or *Fbw7* are found in Tg8 tumors.** Lymphomas from 18 different Tg8 mice were analyzed by cDNA sequencing. Primers were designed to amplify the designated exons of *Notch1* and *Fbw7*. PCR products were sent for sequencing, and the data were aligned with WT as well as mutated *Notch1* and *Fbw7*. Mutations were counted and identified.
(TIF)
