# Supplementary figures and images for: Characterization of Two Distinct Lymphoproliferative Diseases Caused by Ectopic Expression of the Notch Ligand DLL4 on T Cells
Source: PLoS One. 2013 Dec 27;8(12):e84841. doi: 10.1371/journal.pone.0084841 (PMC3874025; doi:10.1371/journal.pone.0084841)

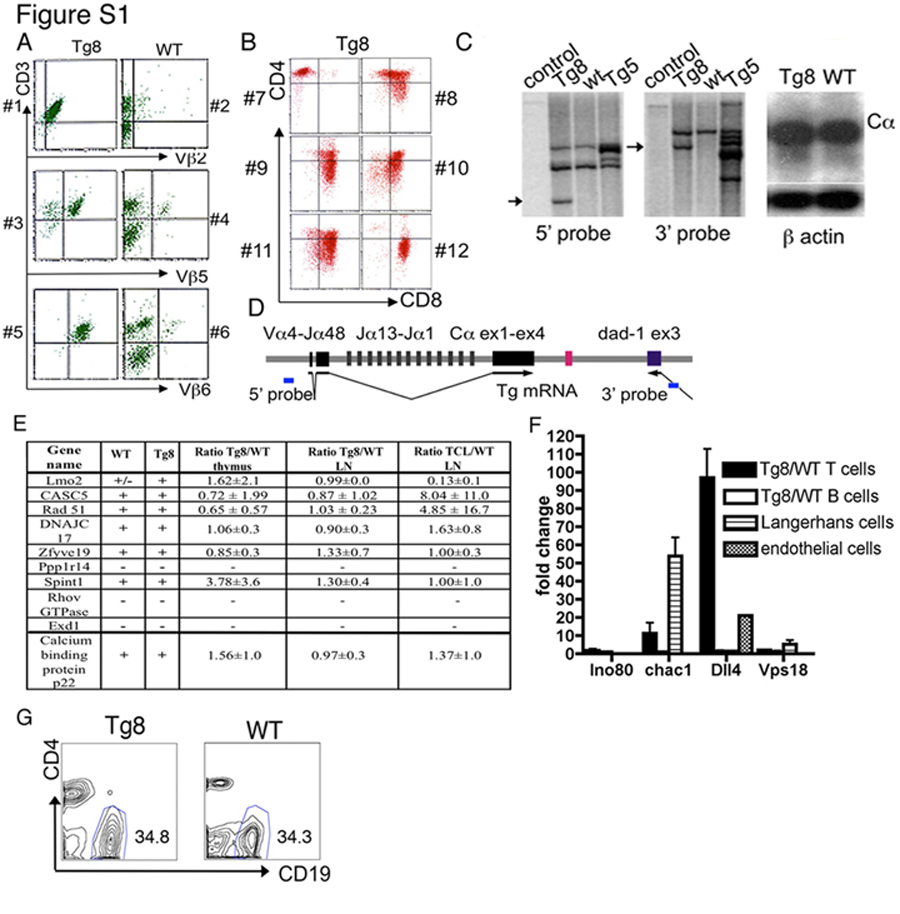

Supplement: Figure S1 — The cellular and genetic characterization of Tg8 tumors. A) Tg8 mice accumulate clonal DP cells. Tg8 tumors are clonal as indicated by dominant usage of one Vβ in each lymphoma (shown are 3 different 4 month-old Tg8 mice (#1, #3, #5, left) comparing to WT littermates (#2, #4, #6, right). B) More than 95% of tumors were CD4+CD8+DP but a small percentage of tumors can display different CD4 and CD8 expression patterns (shown are 6 different Tg8 individuals with lymphomas numbered #7 to #12). C) Tg8 mice have inserted a single copy of the TCRα transgene, and the TCRα is not part of any aberrant transcript. Left panel: Southern blot of DNA extracted from Tg8, WT or Tg5 animals. The probes used are indicated in Figure S1E. The arrows show the 5’ and 3’ flanking sequences in Tg8. Right panel: northern blot probed with a Cα probe and re-probed with β-actin. D) Diagram depicting (to scale) the TCRα construct used to generate lymphoma-prone Tg8 and lymphoma-free Tg5 mice. The position of the main enhancer is indicated by a red box. The position of the 5’ and 3’ probes used in Figure S1D is indicated by blue rectangles below the map. E) Summary of the transcriptional effects of Tg8 insertion at Chromosome 2 for genes located between 100 and 250 kb on either side of the integration site. The thick line between Exd1 and Cbp22 indicates the area closest to the integration site (100 kb on each side). qPCR of the indicated genes was performed, and the ratios of relative transcription level of Tg8 tissue and WT tissue were calculated as listed in the right three columns as thymus, spleen and T lymphoma (TCL). The second and third columns to the left indicate whether the raw expression values of the samples are hardly detectable above the negative control (-), slightly above the negative control (+/-), or clearly above the negative control (+). All changes in expression ratios are non-significant. F) Transcriptional effects of Tg8 insertion on the genes located nearest the integratio [file pone.0084841.s001.tif]

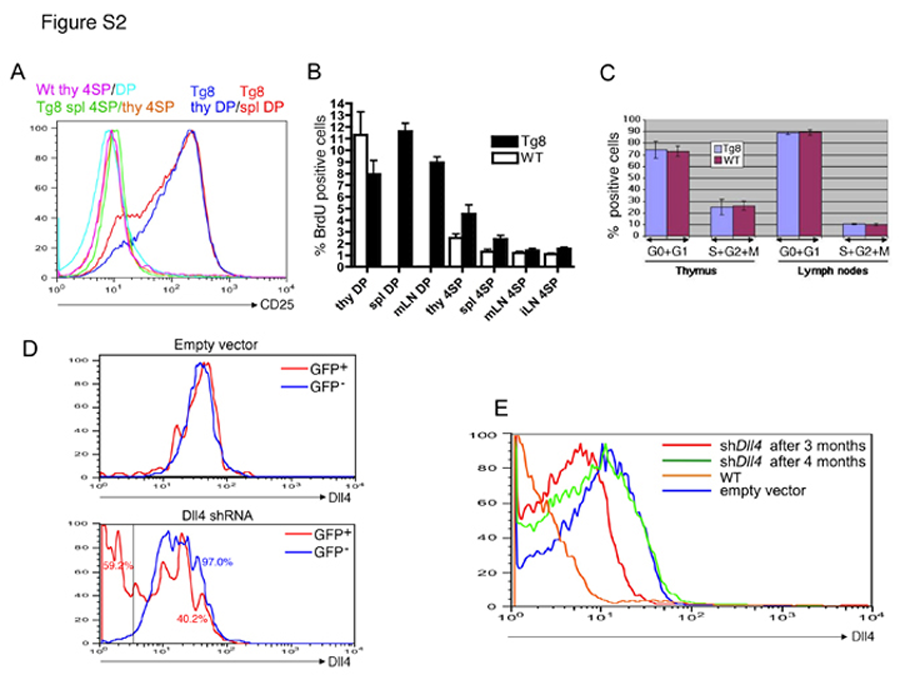

Supplement: Figure S2 — Proliferation profile of Tg8 T cells and the efficiency of knock-down experiments. A) In Tg8 mice, the Notch pathway is preferentially activated in DP cells. Shown is the surface expression of the Notch target gene CD25 in the indicated populations (color-coded). B) Four week-old Tg8 and littermate controls were injected intraperitoneally with BrdU and sacrificed 4 hours later. Thymic, splenic, and mesenteric lymph node cells were surface-stained with anti-CD4 and anti-CD8 antibodies, followed by intracellular staining with anti-BrdU antibody. The panel shows the percentage of BrdU+ cells among the populations indicated in the x axis. Data are represented as mean +/- SEM. C) Propidium iodide staining of permeabilized thymic and lymph node cells from 3 week-old Tg8 and WT littermate (n=3). D) CD4 SP splenocytes sorted from 4 week-old Tg8 were cultured and transduced with either Dll4 shRNA-pQXIP/GFP vector or the same amount of empty pQXIP/GFP vector. On day 3 the cells were analyzed for DLL4 surface expression with two gates on GFP+ and GFP- populations. MFI= 12.9 in GFP- and 28.9 in GFP+ E) 4 week old Tg8 BMs were transduced with either Dll4 shRNA or empty vector, and transferred into RAG1-/- recipients. CD4 SP cells from blood were analyzed for DLL4 expression at 3 and 4 months after BM transfer. Tg8 BM transduced with empty vector was also transferred into RAG1-/- mice as positive controls for lymphoma development; WT BM was transferred into RAG1-/- mice as a negative control for lymphoma development. MFI= 15.3 (empty vector), 12.6 (shRNA after 4 months), 7.3 (shRNA after 3 months), 1.7 (WT). (TIF) [file pone.0084841.s002.tif]

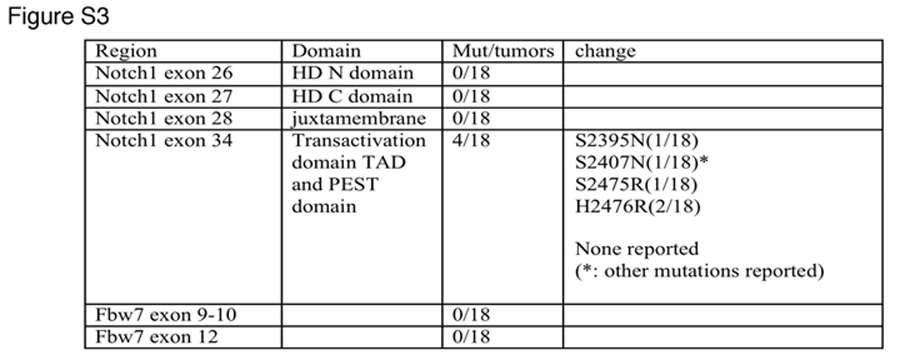

Supplement: Figure S3 — None of the known T-ALL mutations of Notch1 or Fbw7 are found in Tg8 tumors. Lymphomas from 18 different Tg8 mice were analyzed by cDNA sequencing. Primers were designed to amplify the designated exons of Notch1 and Fbw7. PCR products were sent for sequencing, and the data were aligned with WT as well as mutated Notch1 and Fbw7. Mutations were counted and identified. (TIF) [file pone.0084841.s003.tif]
